# Sealing Ability of Alkaline Endodontic Cements versus Resin Cements

**DOI:** 10.3390/ma10111228

**Published:** 2017-10-25

**Authors:** Yu-Yao Teoh, Basil Athanassiadis, Laurence J. Walsh

**Affiliations:** 1 School of Dentistry, The University of Queensland, Herston, Brisbane, QLD 4006, Australia; l.walsh@uq.edu.au; 2 Private Dental Practice, Annerley, Brisbane, QLD 4103, Australia; basildent@bigpond.com

**Keywords:** calcium hydroxide, MTA, alkaline cement, bacterial penetration, endodontic sealer

## Abstract

Penetration of oral bacteria through root fillings leads to their long term failure. Dimensionally stable alkaline cements have been developed. A saliva challenge model was used to compare resistance to bacterial penetration of these alkaline cements to conventional root fillings that combine gutta percha (GP) with epoxy resin sealers. A sample of 140 human roots with single straight canals prepared to standard length and canal size were obturated with mineral trioxide aggregate (MTA) (Nex MTA or MTAmix), with an alkaline calcium hydroxide hard setting cement (Supercal), or with GP and a resin cement (either AH-Plus or Zirmix). Negative control roots were sealed with wax, while positive controls were left open. The test assemblies were gamma sterilised, then the coronal root face was exposed daily to fresh stimulated human saliva diluted in broth. Bacterial penetration was determined by assessing growth in sterile brain-heart infusion (BHI) medium in contact with the root apex. Using Kaplan–Meier survival analysis, in order of performance from highest to lowest: Negative control, Supercal, Nex MTA, Zirmix, MTAmix, GP + AH-Plus, and the positive control. In addition, statistically significant differences were noted between Supercal and AH-Plus, and between the two MTA cements. It can be concluded that alkaline cements, particularly Supercal, can show considerable resistance to bacterial penetration from constant saliva challenge, and provide superior sealing ability in comparison to resin cements. While this property is due mostly to dimensional stability, the release of hydroxide ions could be a contributing factor to impaired bacterial survival, and this aspect should be explored further.

## 1. Introduction

In endodontics, a range of materials are used to fill the root canal of a tooth after chemo-mechanical preparation has been completed. The root-filling material is expected to provide a hermetic seal against microorganisms [1]. It is typically applied in a plastic state which allows the filling of small anatomical intricacies of a tooth, however the change of state from plastic to rigid may be accompanied by a change in dimension [2]. A common approach to filling the root canal uses gutta percha (GP) cones [3] as a core material. These cannot provide a hermetic seal due to the lack of adhesion to dentine [4]. Because root canals are often irregularly shaped, these cones are heated to soften them to improve their adaptation to the canal walls. To help achieve complete obturation in three dimensions and provide a seal for the canal, the GP is combined with a sealer, typically consisting of epoxy resin [2,4]. The entire filling is imperfect because thermo-plasticised GP shrinks upon cooling [5]. Moreover, cohesive fractures and voids are created when the epoxy resin sealer undergoes shrinkage as it polymerises [6,7,8]. Once coronal leakage has occurred, these voids create a pathway along which bacteria may penetrate through the root filling, for example through the sealer, along the walls of the root canal system, or at the interface between the GP core and resin cement [6,8].

An alternative approach to obturation of the root canal would be to employ a single rigid cement material which is dimensionally stable and able to adapt to the canal walls and so obturate the entire root canal space [2]. Alkaline cements that release calcium hydroxide are of potential interest for this purpose, given the known benefits of calcium hydroxide pastes as antimicrobial agents when used to medicate the root canal system [9]. However, current calcium hydroxide-based sealers have poor resistance to bacterial leakage due to adaptability issues with canal walls and cement dissolution [10,11].

Mineral trioxide aggregate (MTA) cements release calcium hydroxide as a reaction product. Rigid or hard-setting calcium hydroxide cements have also been developed without water in their composition to eliminate dehydration and subsequent shrinkage. These are based on the reactions of calcium hydroxide with glycerol. The set cement is a dense rigid material, within which freely available excess calcium hydroxide is trapped which has not been incorporated chemically into the set reaction products. Release of this excess calcium hydroxide can occur when water comes into contact with the surface of the set cement, but contact with water does not result in dissolution of the cement [12]. Release of hydroxide ions from these alkaline cements may exert antimicrobial actions, thereby reducing bacterial penetration along their interface with dentine [9]. The present study was undertaken to compare bacterial penetration through various cements, when faced with continuous exposure to salivary microorganisms in a rich broth environment using the bacterial penetration model described by Torabinajed et al. [13]. The working hypothesis for the study was that alkaline cements provide better resistance against bacterial penetration than the combination of GP with an epoxy resin cement.

## 2. Materials and Methods

### 2.1. Collection and Preparation of Teeth

Single-canal teeth with roots of comparable size and length were selected from a pool of extracted teeth collected from an oral surgery clinic with the approval of the institutional ethics committee (Approval code #1311). Roots were inspected for cracks via transillumination with a handheld transilluminator (Microlux™, AdDent, Danbury, CT, USA) and discarded if a crack was found. The selected teeth were decoronated at the cemento-enamel junction, and the cut surface was flattened with a diamond bur to give samples of consistent length. The root canals were assessed for apical patency with a #8 K-file (Dentsply Maillefer, Ballaigues, Switzerland). Any teeth with calcifications or blockages were discarded.

A total of 140 teeth were divided into 7 groups of 20 each by random assignment. The root canals were prepared with nickel-titanium rotary instruments, (ProTaper Next™, Dentsply Maillefer, Ballaigues, Switzerland) to size X3 with an ISO #30 apical preparation and variable taper. The root canals were irrigated alternately with 1% *w*/*v* sodium hypochlorite (Endosure Hypochlor 1% Solution™, Dentalife, Melbourne, Australia) and 15% *w*/*v* ethylenediaminetetraacetic acid with 0.85% *w*/*v* cetrimide (Endosure EDTA/C 15% Solution™, Dentalife) using syringes with side-vented needles. After a final irrigation with EDTA/C, the canals were dried with paper points. The roots were then stored in saline at 22 °C until used.

### 2.2. Placement of Materials

A total of five different materials (Table 1) were used to obturate the canals, with two groups serving as controls.

Both AH-Plus™ and Zirmix™ resin cements were used in conjunction with GP points to obturate the canal using the lateral condensation technique. Zirmix is a modification of the formula of an older product, AH26™, with only the bismuth trioxide replaced with zirconium dioxide to prevent staining of roots over time. A ProTaper Next™ X3 GP point (Dentsply Maillefer, Ballaigues, Switzerland) with a #30 apical size and variable taper was used as the master cone, followed by medium and fine accessory points placed with the aid of a finger spreader.

Nex MTA™ (grey MTA), MTAmix™ (white MTA) and Supercal™ were used as single obturating materials. These materials were hand-mixed according to the manufacturer’s instructions, then injected into the canal using a syringe with a fine nozzle under positive pressure and back-flowed from the apex. Complete obturation of all roots was confirmed using digital radiographs taken at 65 kV and 7 mA, with an exposure time of 0.16 s. Voids present in the obturation were eliminated by compaction of the cement with an endodontic plugger.

As positive controls, 20 roots had their canals left open to ensure bacterial penetration to the apical region. A further 20 roots were sealed at the coronal surface and at the apical third with molten red boxing wax, and served as a negative control to assess possible contamination in the model system used.

### 2.3 Mounting of Samples

**Figure 1:**
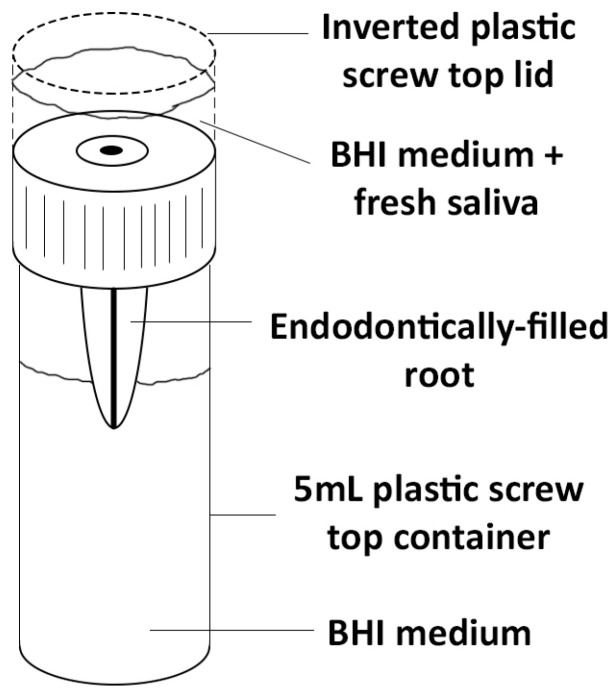
Schematic diagram of the experimental model.

Pink nail varnish was used to coat the outer surface of all roots, other than for the coronal surface and the apical third. This was done to seal any unseen lateral canals or microscopic cracks. The roots were mounted in 5 mL specimen containers with an additional inverted lid which served as a reservoir for the growth medium containing bacteria from saliva. Holes were drilled into the lids and the roots secured in place through these holes with red boxing wax, as employed in previous studies [11,13,14]. The specimen assemblies (Figure 1) were placed into an air-tight container and gamma irradiated with 25.4 kGy at a commercial gamma sterilisation facility (Steritech, Queensland, Australia).

Sterile brain-heart infusion (BHI) medium was prepared from stock powder (BBL Brain Heart Infusion™, Becton Dickinson, Franklin Lakes, NJ, USA) and loaded into the lower reservoirs of the sterilised specimen assemblies within a biosafety hood. The samples were then monitored over a 24 h period, to exclude those with environmental contamination.

### 2.4 Exposure to Bacteria

Fresh human saliva was collected daily for 90 consecutive days from a single healthy donor with the aid of paraffin wax (Saliva Test Buffer™ kit, GC Corporation, Tokyo, Japan) to stimulate saliva flow. A range of measures were undertaken to ensure consistency in the samples. Firstly, the samples were collected at the same time each day (6:00 p.m.). The subject did not use antibiotics or antimicrobial mouthwashes during the study, and followed an identical protocol for oral hygiene each day during the study. There was no oral hygiene undertaken in the 10 h prior to saliva collection, nor any foods or drinks in the 5 h prior to collection.

Immediately after collection, the saliva was mixed with sterile BHI medium in a 1:5 dilution, and the mixture placed into the uppermost inverted lid to expose the coronal surface of the roots to bacterial contamination. This mixture of saliva and BHI was replaced with a fresh inoculum each day for a total of 90 days or until bacterial penetration was identified. The daily inoculum was mixed thoroughly before application to the samples to ensure that the microbial challenge was the same. On each day, samples were taken of the salivary inoculum and were added to BHI broth, to check for growth using turbidity as a visual readout. The BHI salivary inoculum model was chosen to mimic an exposed root canal in the oral cavity. Prior studies using this model in the laboratory had shown that the salivary inoculum contained a diverse range of oral bacteria, as shown by Next-Gen sequencing [15].

**Figure 2:**
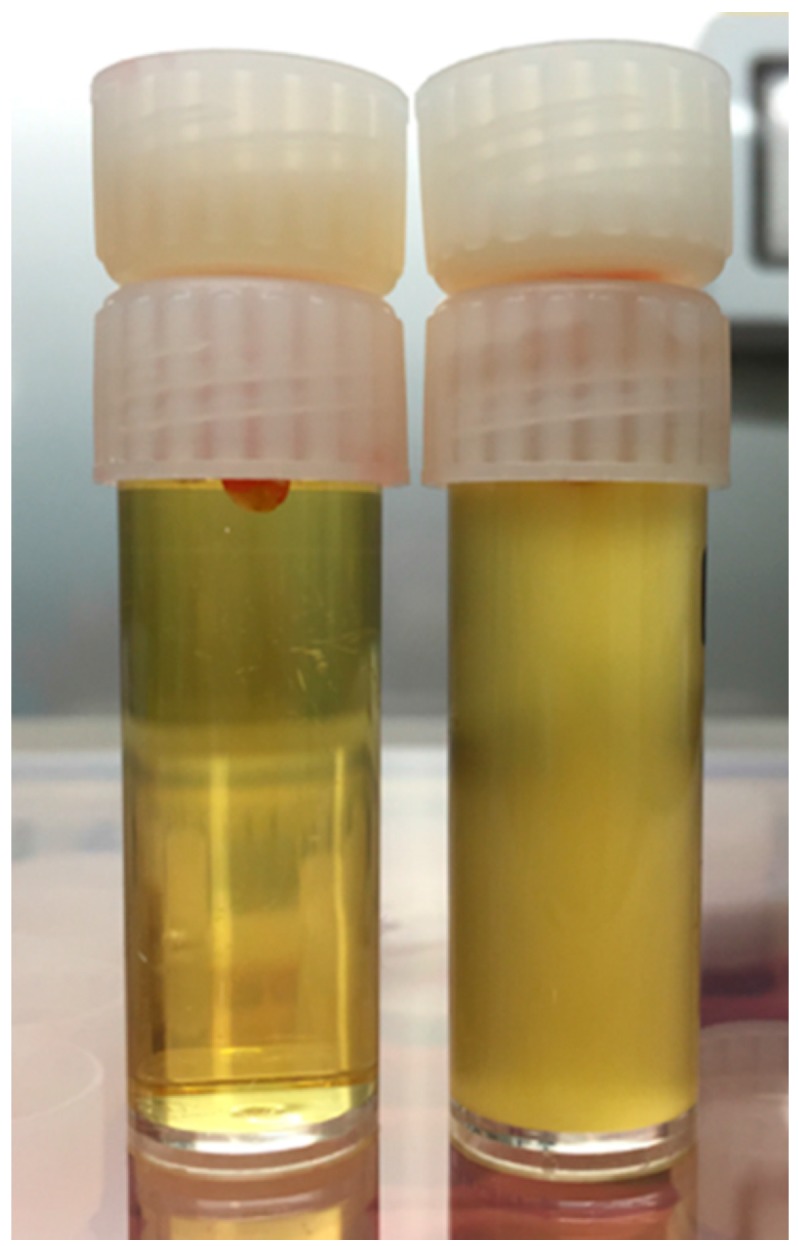
BHI medium in clear (left) and turbid states (right).

The assemblies were kept in a fully humid environment at 37 °C in an incubator. Bacterial penetration was identified by turbidity in the BHI broth which contacted the apex of the tooth (Figure 2). The time (in days) at which bacterial penetration occurred was used to plot survival curves.

### 2.5 Statistical Analysis

To assess bacterial penetration for the different materials, Kaplan–Meier survival curves were plotted. The median survival time (in days) to reach the point of bacterial penetration through 50% of samples was calculated for the various materials. Log-rank tests were used to assess the statistical significance of differences between materials, using a threshold of *p* < 0.05.

## 3. Results

All 20 positive controls consisting of roots with open canals failed by the second day. None of the test materials completely resisted bacterial penetration. Ranked from best performing to worst, Supercal™ had five surviving samples at the end of 90 days, followed by Nex MTA™ with two, and Zirmix™ and MTAmix™ each with one. All AH-Plus™ samples leaked within 48 days.

**Figure 3:**
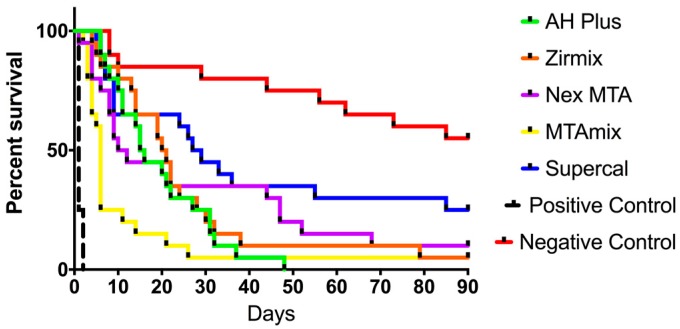
Kaplan–Meier survival curves for endodontic materials subjected to challenge from salivary bacteria over 90 days.

Kaplan–Meier survival curves are shown in Figure 3. Median survival time (in days) for the various materials and corresponding P values of differences between materials are shown in Table 2 and Table 3, respectively. Supercal™ was significantly better at resisting bacterial penetration than all other materials with the highest median survival of 28 days when exposed to salivary bacteria. Comparing the performance of materials of similar composition, within the epoxy resin cements Zirmix™ performed better than AH-Plus™ in terms of median survival, but this difference was not statistically significant (*p* = 0.4136). For the MTA cements, Nex MTA™ had a higher median survival than MTAmix™, and this difference was statistically significant (*p* = 0.0269) (Table 3).

## 4. Discussion

The results of this study show that under the challenge of continuous exposure to salivary microorganisms in a nutrient-rich medium, none of the endodontic materials could completely prevent bacterial penetration through the root filling. The average time for microorganisms to pass through the root filling to reach the apex varied widely, with Supercal™ giving the longest time of four weeks. The time required for bacterial penetration was lower for epoxy resin cements than either for MTA or Supercal™. This may reflect the impact of polymerisation shrinkage during the setting reaction of epoxy resins [6,7].

While both epoxy resin cements that were used with GP in the lateral condensation technique had similar composition, the performance of Zirmix™ was superior to that of AH-Plus™. Like AH26™, Zirmix™ is a powder-liquid system and was included in this study to assess its sealing ability against AH-Plus™ as an alternative non-staining epoxy resin cement. Zirmix is a modification of the formula of an older product, AH26™, with only the bismuth trioxide replaced with zirconium dioxide to prevent staining of roots over time. The results from this study are consistent with a past study that showed an inferior sealing ability of AH Plus™ compared to AH26™ [16]. Zmener et al. described the faster setting and more pronounced shrinkage stress of AH-Plus™ as being responsible for earlier de-bonding from dentine walls, hence greater bacterial penetration. Unlike AH26™, AH-Plus™ contains silicone oils that could prevent wetting of the root-canal wall and impair adhesion to humid dentine [16].

The present results revealed differences between the two MTA cements. When we examined samples of the cement powders using light microscopy, MTAmix contained larger and coarser particles than Nex MTA™. In a hand-mixed material, having larger particles may lead to greater porosity within the set cement, which then influences bacterial penetration. An additional observation was that all roots treated with Nex MTA™ showed intense grey discolouration. This a well-known issue with grey MTA cements [17]. No such changes were seen with MTAmix™, Supercal™, or the epoxy resin materials.

Some aspects of the continuous bacterial leakage model described by Torabinejad et al. [13] have been questioned in the literature, such as the ability of sticky wax to provide completely hermetic seal between the components of the model [18]. While it is possible that some leakage could occur at the sticky wax interface [18], this is not the only pathway, since leakage may also occur between the root filling and the canal wall. The present study used red boxing wax instead of sticky wax to seal the components to overcome any issues with brittleness of the wax, and also included controls to assess whether such leakage occurred. Nevertheless, as there was no thermal cycling it is not possible to extrapolate the bacterial penetration dynamics directly to the clinical situation, e.g., in terms of saliva contamination of a fractured tooth which has the root filling material exposed. Rather, the present study reveals differences in the performance of materials under the same testing conditions which provide information regarding their ability to resist bacterial contamination.

The results of the present study show that hard-setting alkaline cements that are dimensionally stable exhibit superior resistance to bacterial penetration. It is possible that the higher resistance of alkaline cements (i.e., MTA and Supercal™) to bacterial penetration could also be aided by the antimicrobial effects from calcium hydroxide released onto the root canal walls on contact with water. Hydroxide ions when present at high levels have antibacterial actions [12,19]. Further investigations are necessary to assess the performance of calcium hydroxide-containing materials used as a hard-setting cement and the possibility of its use as a single agent for root filling.

## 5. Conclusions

No root filling material was able to completely prevent bacterial penetration when faced with a constant challenge from salivary microorganisms. Within the timeframe and limits of this study, hard-setting alkaline cements based on MTA or calcium hydroxide show greater resistance to bacterial penetration than epoxy resin cements. However, the possible contribution of antimicrobial effects from hydroxide ion release should be investigated further.

## Figures and Tables

**Table 1:** Experimental groups.

Material	Manufacturer	Description
AH-Plus™	Dentsply Maillefer, Ballaigues, Switzerland	A non-staining epoxy resin two paste sealer.
Zirmix™	Ozdent, Castle Hill, Sydney, Australia	A non-staining epoxy resin power/liquid sealer
Nex MTA™	GC Corporation, Tokyo, Japan	A grey MTA cement
MTAmix™	Ozdent, Castle Hill, Sydney, Australia	A white MTA cement
Supercal™	Ozdent, Castle Hill, Sydney, Australia	A novel calcium hydroxide alkaline cement with a glycerol-based solvent.

**Table 2:** Median survival in days for endodontic obturation materials and controls.

Material	Median Survival (Days)
AH Plus™	15.5
Zirmix™	20.5
Nex MTA™	11.0
MTAmix™	6.0
Supercal™	28.0
Positive control	1.0

**Table 3:** Summary of differences between groups.

Material	AH Plus	Zirmix	Nex MTA	MTAmix	Supercal	Positive Control	Negative Control
AH Plus	-	0.4136	0.2652	0.0144	0.0192	0.0001	0.0001
Zirmix	-	-	0.8156	0.0083	0.0771	0.0001	0.0001
Nex MTA	-	-	-	0.0269	0.1783	0.0001	0.0002
MTAmix	-	-	-	-	0.004	0.0001	0.0001
Supercal	-		-	-	-	0.0001	0.0196
Positive Control	-	-	-	-	-	-	0.0001
Negative Control	-	-	-	-	-	-	-

*p* values were determined from Log-Rank tests.
